# Rapid and Sensitive Detection of Sulfamethizole Using a Reusable Molecularly Imprinted Electrochemical Sensor

**DOI:** 10.3390/foods12081693

**Published:** 2023-04-19

**Authors:** Jie Kong, Xiaoli Xu, Yixin Ma, Junjian Miao, Xiaojun Bian

**Affiliations:** 1 College of Food Science and Technology, Shanghai Ocean University, Shanghai 201306, China; m200300916@st.shou.edu.cn (J.K.); xlxu981023@163.com (X.X.);; 2 Laboratory of Quality and Safety Risk Assessment for Aquatic Product on Storage and Preservation (Shanghai), Ministry of Agriculture, Shanghai 201306, China; 3 Shanghai Engineering Research Center of Aquatic-Product Processing & Preservation, Shanghai 201306, China

**Keywords:** electrochemical sensor, molecularly imprinted polymers, conductive polymer, antibiotic, sulfamethizole, thiophene

## Abstract

Efficient methods for monitoring sulfonamides (SAs) in water and animal-source foods are of great importance to achieve environmental safety and protect human health. Here, we demonstrate a reusable and label-free electrochemical sensor for the rapid and sensitive detection of sulfamethizole based on an electropolymerized molecularly imprinted polymer (MIP) film as the recognition layer. To achieve effective recognition, monomer screening among four kinds of 3-substituted thiophenes was performed by computational simulation and subsequent experimental evaluation, and 3-thiopheneethanol was finally selected. MIP synthesis is very fast and green, and can be in situ fabricated on the transducer surface within 30 min in an aqueous solution. The preparation process of the MIP was characterized by electrochemical techniques. Various parameters affecting MIP fabrication and its recognition response were investigated in detail. Under optimized experimental conditions, good linearity in the range of 0.001−10 μM and a low determination limit of 0.18 nM were achieved for sulfamethizole. The sensor showed excellent selectivity, which can distinguish between structurally similar SAs. In addition, the sensor displayed good reusability and stability. Even after 7 days of storage, or being reused 7 times, higher than 90% of the initial determination signals were retained. The practical applicability of the sensor was also demonstrated in spiked water and milk samples at the nM determination level with satisfactory recoveries. Compared to relevant methods for SAs, this sensor is more convenient, rapid, economical, and eco-friendly, and had comparable or even higher sensitivity, which offered a simple and efficient method for SA detection.

## 1. Introduction

Sulfonamides (SAs) are a class of broad-spectrum synthetic antibacterial and anti-inflammatory antibiotics, including sulfacetamide, sulfadiazine, and sulfamethizole (SMZ). SAs are widely used for the prevention and treatment of human and animal infections as well as growth promotion in animal husbandry due to their high efficacy and low cost [1]. However, the excessive use of SAs has led to SA residue contamination of various animal-derived foods, and the natural environment (e.g., water bodies and soil), which causes serious health issues in humans, including drug resistance, allergic reactions, enteric flora disturbance as well as potentially carcinogenic, teratogenic, and mutagenic effects [2,3]. Therefore, the determination of SA residues in water and animal by-products (e.g., milk) becomes very important.

At present, various analytical methods have been reported for detecting SAs, including high-performance liquid chromatography (HPLC) [4], ultra-high-performance liquid chromatography–mass spectrometry (UPLC–MS) [5], liquid chromatography–mass spectrometry (LC–MS) [6], capillary electrophoresis [7], immunoassays [8], surface-enhanced Raman spectroscopy (SERS) [9], and using electrochemical sensors [10,11]. Among them, electrochemical methods offer the benefits of elevated sensitivity, accuracy, convenient operation, cheap instrumentation, facile integration, and portability [12].

To fabricate an efficient electrochemical sensor, among the most important aspects is choosing a suitable recognition element (receptor). Molecularly imprinted polymers (MIPs) are tailor-made biomimetic supramolecular receptors that recognize and bind target molecules with high affinity and selectivity, comparable to those of antibodies [13]. They possess many unique features such as easy preparation, cost-efficient, good stability, and reusability [14]. Due to these superior characteristics, MIPs as recognition elements have been combined with various electrochemical techniques for detecting various small molecules such as antibiotics [15], pesticides [16], and mycotoxins [17]. The efficient recognition of MIPs towards the analyte (often the template) depends largely on the interaction (covalent or non-covalent) between a template and a functional monomer [18,19]. For better template removal, non-covalent interactions such as hydrogen bonds are preferable. Currently, frequently used functional monomers for MIPs synthesis include 3-aminophenylboronic acid [20], resorcinol [21], methacrylic acid [22], 4-aminothiophenol [23], chitosan [24], and acrylamide [25]. What these monomers have in common is that they have special functional groups that could afford sufficient binding sites with the template through non-covalent interaction (e.g., hydrogen bonds, electrostatic, and/or π–π interactions). Unfortunately, most of them are non-conductive or poorly conductive after polymerization, which is unfavorable for electron transfer, and thus may affect the sensitivity of the sensor. To address this issue, various nanomaterials were introduced for sensing interface construction. However, the synthesis of nanomaterials is normally tedious, and time consuming. Additionally, the use of nanomaterials may increase the sensors’ cost and differences between batches. In this case, it is difficult to achieve a simple, fast, and low-cost determination.

**Scheme 1:**
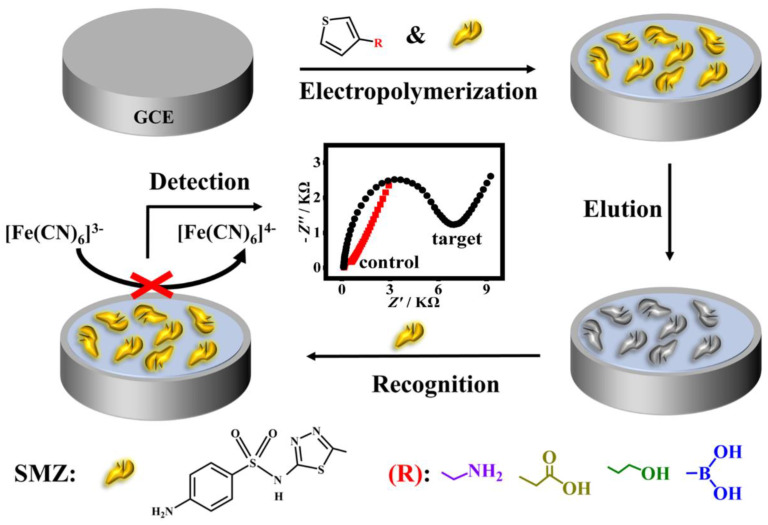
Schematic diagram of the preparation of a MIP-based impedance sensor for sulfamethizole (SMZ) detection.

Herein, polythiophene derivatives, having the advantages of high environmental stability and good water solubility [26], were chosen as the polymer matrices for imprinting SMZ as a model of SAs. To establish hydrogen bond interaction between the SMZ template and the monomer, four kinds of thiophene substituted at the 3-position by different functional groups including carboxylic acid, amino, boric acid, and hydroxyl group were rationally chosen as monomer candidates. The quantum chemical calculation was first performed to analyze the interactions between the SMZ molecule and several monomers. Subsequently, experiments were carried out to assess the recognition effect of the four imprinted polymers based on the imprinting factor (IF), and 3-thiopheneethanol was eventually used. On this basis, a conductive molecularly imprinted polymer film-based impedimetric sensor was developed for simple, rapid, sensitive, and low-cost determination of SMZ. As shown in Scheme 1, the MIP film was in situ deposited on a glassy carbon electrode (GCE) surface by one-step electro-copolymerization of the SMZ template and one of the four 3-substituted thiophene monomers followed by elution of the template. The as-synthesized MIP films with specific imprinted sites that are complementary with SMZ in size, shape, and functionality are expected to selectively recognize SMZ. Upon rebinding with the non-conductive SMZ, the electron transfer between the electrode surface and the [Fe(CN)_6_]^3−/4−^ redox probe in the electrolyte solution was blocked, resulting in a significant increase in film impedance. In this way, the target SMZ could be quantified according to the impedance change. The processes of imprinting and recognition were characterized by electrochemical impedance spectrum (EIS) and cyclic voltammetry (CV). A series of experimental parameters affecting the MIP preparation and its recognition were investigated and optimized. Under optimized conditions, the sensor showed good performances with a wide linear range, a low determination limit as well as excellent selectivity, reusability, and stability. In addition, the sensor had good applicability in spiked water and milk samples. Compared to most reported MIP-based electrochemical sensors for detecting SA antibiotics [27,28,29], this assay is more convenient, rapid, and sensitive, with a comparable or even lower limit of detection (LOD, 0.18 nM), and a much quicker preparation process (<30 min) for the sensing interface.

## 2. Experimental Section

### 2.1. Reagents

SMZ, 3-thiopheneboronic acid (TBA), sulfanilamide (SA), sulfathiazole (ST), 3-thienylmethylamine (TM), thiophene-3-ethanol (TE), 3-thiopheneacetic acid (TAA), and methanol (MeOH) were bought from Sigma-Aldrich. Acetic acid (HAc), sodium dodecyl sulfate (SDS), trichloroacetic acid, and cetyltrimethylammonium bromide (CTAB) were obtained from BBI Life Sciences Corporation (Shanghai, China).

### 2.2. Apparatus and Measurements

Electrochemical experiments were performed using a CHI 660E (CH Instruments, China) workstation with a standard three-electrode system. A GCE (3 mm in diameter), a saturated calomel electrode (SCE), and a platinum sheet served as the working, reference and counter electrodes, respectively. CV measurements were performed in 0.1 M KCl containing 5 mM K_3_[Fe(CN)_6_]. The EIS was recorded in 0.1 M KCl containing 1 mM K_3_[Fe(CN)_6_] and 1 mM K_4_[Fe(CN)_6_], with an open circuit voltage in a frequency range of 0.1–100,000 Hz using an amplitude of 5 mV. The EIS response towards the analyte was represented as the relative change in charge transfer resistance (*R*_ct_) [∆*R/R*(Ω)], calculated according to the following equation:

Δ*R/R* = (*R*_cta_ − *R*_ctb_)/*R*_ctb_

where *R*_ctb_ and *R*_cta_ are the values of *R*_ct_ before and after SMZ rebinding, respectively [30].

### 2.3. Preparation of the MIP Film-Modified Electrode (MIP/GCE)

The GCE was polished with 0.05 μm Al_2_O_3_ aqueous slurry to a mirror-like surface for subsequent modification. One-step electrochemical polymerization was performed by CV at a scan rate of 100 mV/s in the potential range of −0.6–1.0 V in 5 mL of a solution in the presence of one of the four monomers (8 mM, TBA, TM, TAA, or TE) and the SMZ template (25 μM). The produced modified electrode was denoted as PTE + SMZ/GCE. To wash off SMZ from the electro-copolymerized film on the GCE surface, 1 mM CTAB dispersed in 36% HAc (CTAB/HAc) was used as an eluent for 20 min, and the modified electrode after removal of SMZ was defined as a MIP/GCE. As a control, the same polymerization method was used to prepare the non-imprinted polymer (NIP)-modified electrode (NIP/GCE) without adding SMZ.

### 2.4. Optimization of Experimental Conditions

Various experimental parameters relative to the MIP preparation and its recognition, including template concentration, electropolymerization cycles, elution conditions (eluents and time), oscillation speed, and pH in recognition were optimized. The optimum eluents and elution time were based on the final *R*_ct_ value after elution. The smaller the *R*_ct_ value, the cleaner the elution. The other optimal experimental parameters were based on the EIS response of the sensor to 0.1 μM SMZ.

### 2.5. Quantitative Determination of SMZ

Under optimized experimental conditions, the as-prepared MIP/GCE was incubated with 300 μL of 10-fold serial diluted SMZ solutions for 30 min. The formed modified electrode (named MIP-SMZ/GCE) was washed with water and dried at room temperature for immediate EIS measurements.

### 2.6. Preparation of Spiked Water and Milk Samples

The water sample was taken from a lake on our campus. The milk (Yili Brand, China) was purchased from the local supermarket. For water, no sample pretreatment was performed. For milk, to reduce the interference of matrices (e.g., fat and protein), the following treatments were applied according to previous studies [31,32]. First, 1 mL of milk was added to 9 mL of a phosphate-buffered solution (0.01 M, pH = 7.0) containing 4% trichloroacetic acid. After vortex for approximately 5 min, the mixture was filtered through a 0.22 μm membrane. The resulting filtrate was collected for further use. The water and milk samples were prepared by a standard addition method to final concentrations of 1, 10, and 100 nM. As a control, phosphate-buffered solution (0.01 M, pH = 7.0) was added in place of SMZ.

### 2.7. Quantum Chemical Calculations

The details for quantum chemical calculations are presented in the Supplementary Materials. The optimized structures for the SMZ molecule and several monomers are shown in Figure S1.

## 3. Results and Discussion

### 3.1. Analysis of Binding between the Template Molecule and Several Monomers

**Figure 1:**
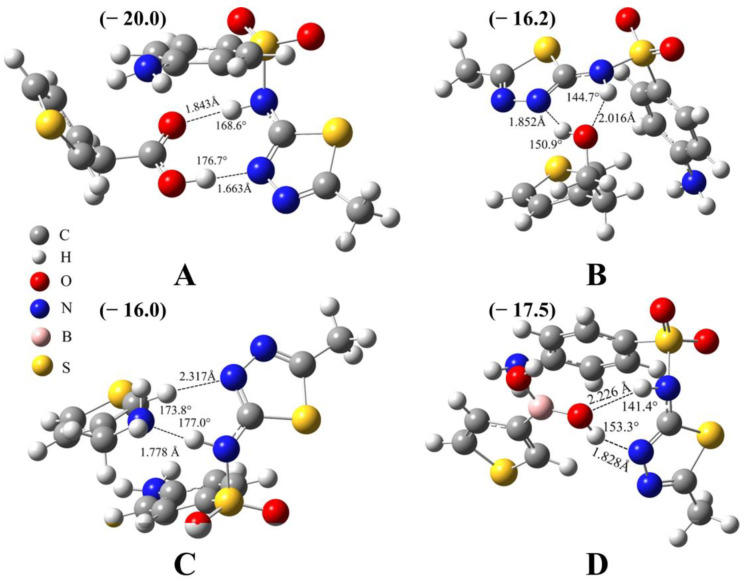
Optimized 3D complex structures between sulfamethizole and (**A**) 3-thiopheneacetic acid, (**B**) 3-thiopheneethanol, (**C**) 3-thiophenemethylamine, and (**D**) 3-thiopheneboronic acid. The presence of hydrogen bonds is indicated by the light black dashed lines. The values near the hydrogen bonds are the bond lengths and bond angles, and values in brackets are binding energies; unit: kcal/mol. Level of theory: PWPB95(D3-BJ)/def2-QZVPP//B97-3c.

The binding between the template and one of the four monomers is analyzed via quantum chemical calculations. The lowest-energy structures and the corresponding binding energy, bond length, and bond angle are shown in Figure 1. It can be seen that all the binding energies are quite large, ranging from −16.0 to −20.0 kcal/mol, indicating the strong interaction between each monomer and the SMZ molecule. For the TAA-SMZ system (Figure 1A), there are two hydrogen bonds: N-H⋯O and O-H⋯N. For the N-H⋯O hydrogen bond, the bond length is 1.843 Å and the bond angle is 168.6°. For the O-H⋯N hydrogen bond, the bond length is 1.663 Å and the bond angle is 176.7°. Both of them are classical hydrogen bonds, and their bond energies are quite large, which is the main reason for their strong combination, indicating by quite low binding energy of −20.0 kcal/mol. In addition to the above-mentioned binding mode, there are other low-lying binding modes for the TAA-SMZ system (Figure S2B,C), which also contain N-H⋯O and O-H⋯N hydrogen bonds. The difference is only that the bond length, bond angle, and the relative orientation of some weak interaction fragments have changed to a certain extent.

The TE-SMZ system also contains N-H⋯O and O-H⋯N hydrogen bonds (Figure 1B). According to the bond length and bond angle data given in the figure, it can be seen that they are all typical hydrogen bonds and have strong interactions. This is the main reason for the strong combination of the two components. Since the template molecule has multiple binding sites, there are also other binding modes, including the O-H⋯N hydrogen bond in Figure S2E and the N-H⋯O hydrogen bond in Figure S2F. However, the binding strength is weaker and their energies are slightly higher than that of the lowest-energy structure in Figure 1B.

The TM-SMZ system contains a N-H⋯N classical hydrogen bond and a C-H⋯N non-classical hydrogen bond (Figure 1C). Two other binding modes are shown in Figure S3B,C, with slightly higher energies than Figure 1C. Figure S3B has two N-H⋯N hydrogen bonds. From the bond length and bond angle data given in the figure, it can be seen that one is stronger and the other is weaker. As for Figure S3C, there is only one classical hydrogen bond, which exists between two amino groups (-NH_2_).

As for the TBA-SMZ system (Figure 1D), it contains O-H⋯N and N-H⋯O hydrogen bonds. The bond length and bond angle of the former are 1.828 Å and 153.3°, respectively, and its effect is strong, while the bond length and bond angle of the latter hydrogen bond is 2.226 Å, 141.3°, respectively, and its effect is relatively weak. Since there are two hydrogen bonds, the interaction between the two components is quite strong. Figure S3E,F show two other binding modes, both of which contain two hydrogen bonds. Therefore, they are very close in energy to Figure 1D, within 2.0 kcal/mol.

In summary, there are multiple binding modes between several monomers and template molecules, and they all contain one or two classical hydrogen bonds, as well as other types of secondary interactions. This is the structural basis for a strong interaction between the template and monomers. The existence of hydrogen bonding, especially strong hydrogen bonding should be an important idea for selecting monomers. However, due to the complexity of the specific circumstances of the experiment, the final results need to be verified experimentally. Based on this, these four monomers were all chosen to carry out further experiments to find an optimal system with a better recognition effect.

### 3.2. Monomer Screening

**Figure 2:**
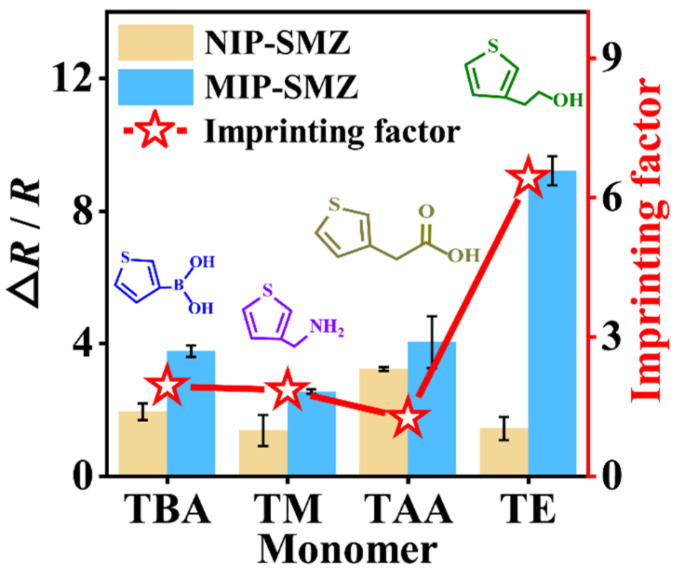
EIS responses (bar charts) and imprinting factors (line charts) of several MIPs prepared with different monomers towards target sulfamethizole (SMZ, 0.1 μM).

Firstly, to evaluate whether these monomers could be used for SMZ imprinting, the EIS was used to characterize the respective imprinting processes. The diameter of the Nyquist plots equals the charge-transfer resistance (*R*_ct_) by fitting the plots using the Randles equivalent circuit (Figure S4), where *R*_s_ is the solution resistance, *R*_ct_ is the charge transfer resistance of the solution, *C*_dl_ is the double layer capacitance, and *Z*_W_ is the Warburg element [33,34]. As shown in Figure S5A–D, for all the monomers, the impedance value of each electro-copolymerized film formed with SMZ was much larger than those formed without SMZ, implying the successful embedment of the template molecules in the polymer matrices. After the step of elution, the impedance value of each formed MIP film was significantly reduced. The results suggest that all four monomers are feasible for SMZ imprinting. Next, the recognition capability of each MIP and NIP towards SMZ (0.1 μM) was studied with the EIS. It can be seen from Figure S5E–H that all the impedance values increased. The corresponding EIS response can be found in Figure 2 (bar charts, left axis). To screen an optimal monomer, the respective imprinting effect was assessed based on IF, which was calculated according to the EIS response of the MIP versus the NIP toward SMZ. As can be seen from Figure 2 (line chart, right axis), the highest IF (6.41) was achieved by the TE monomer, though its binding energy with the SMZ is not the strongest. Hence, TE was ultimately used as the functional monomer for the fabrication of the SMZ-templated MIP film.

### 3.3. Characterization of the Imprinting and Recognition Processes for SMZ

**Figure 3:**
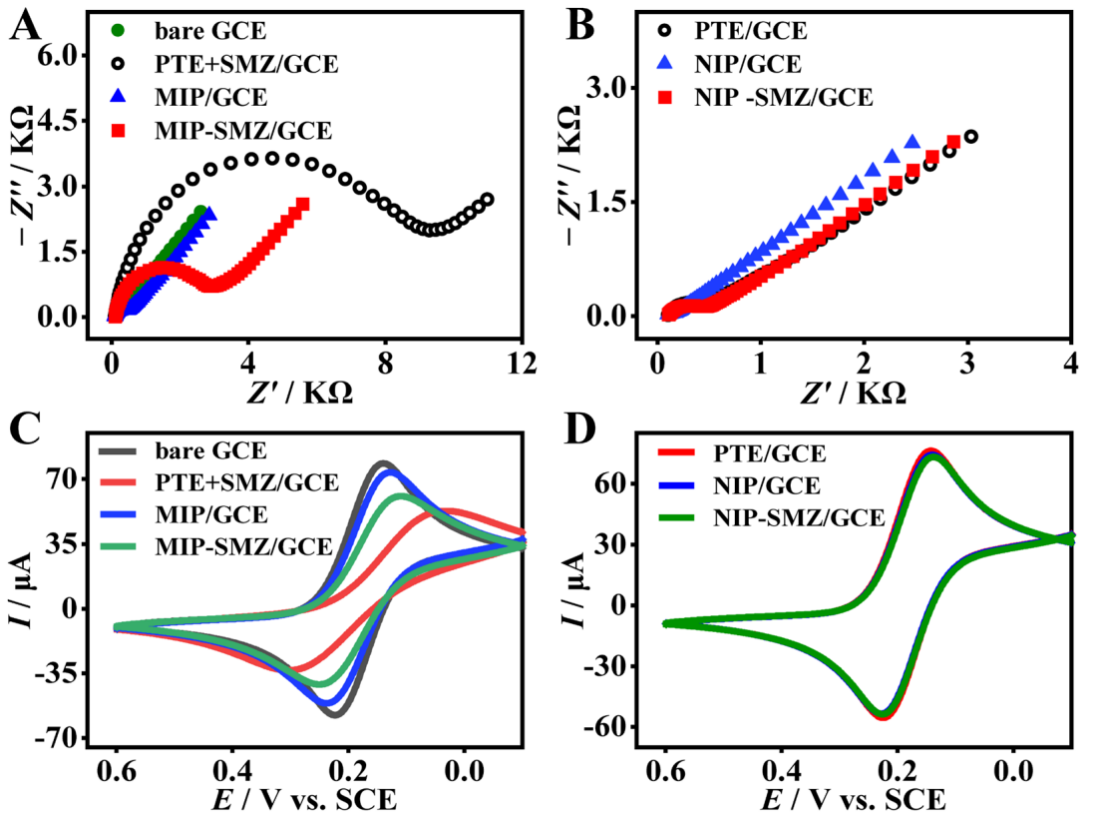
Electrochemical characterization for the fabrication process of the MIP film. (**A**,**B**) EIS and (**C**,**D**) CV curves for different modified glass carbon electrode (GCE).

The processes of SMZ imprinting and recognition was characterized by both the EIS and CV. Figure 3A,B depict the Nyquist plots of different modified electrodes. The bare GCE (Figure 3A, solid circles) exhibits almost a straight line, suggesting a rapid electron transfer on the GCE surface. After the electropolymerization of the TE monomer and the SMZ template (PTE + SMZ/GCE), the semicircle diameter (Figure 3A, hollow circle) increased considerably with a large *R*_ct_ value of ca. 9.9 KΩ, which is 23-fold higher than that of the PTE film alone (PTE/GCE, approximately 430 Ω, Figure 3B, hollow circles). The results showed that the SMZ template molecules were entrapped into the polymer matrices. After eluting the template with CTAB/HAc, the semicircle diameter decreased substantially. The *R*_ct_ value of the formed MIP film (approximately 263.8 Ω Figure 3A, solid triangle) is very close to that of the PTE film, which implies an effective elution of the SMZ template. In addition, the lower *R*_ct_ value indicates a faster electron transfer rate at the MIP film/electrolyte interface, which helps the sensitivity of the label-free sensor. When the MIP film was incubated in SMZ (0.1 μM) for 30 min, the semicircle diameter of the formed MIP-SMZ/GCE (Figure 3A, solid square) amplified significantly. The *R*_ct_ value of the MIP-SMZ/GCE (ca. 2346 Ω) was 9-fold higher than that of the MIP/GCE (ca. 268.3 Ω). However, the impedance of the NIP-SMZ/GCE (Figure 3B, solid square) shows a very tiny increase compared with that of the NIP/GCE (Figure 3B, solid triangle), signifying a negligible non-specific adsorption of the NIP film. The above result suggested that the MIP film had great recognition capability toward the SMZ.

Figure 3C,D shows the CV curves of different modified electrodes as discussed above. Clear redox peaks were found in the CV curves of the bare GCE (Figure 3C, gray line) and PTE/GCE (Figure 3D, red line). After the electro-copolymerization, the PTE + SMZ/GCE (Figure 3C, red line) showed a significant increase in peak potential difference (Δ*E*_p_) and an obvious decrease in the peak current (*I*_p_) compared with that of the PTE/GCE. The result confirmed the successful doping of the SMZ template in the polymer matrices. When the SMZ was eluted, the redox peaks of the formed MIP/GCE (Figure 3C, blue line) were more symmetrical and the currents were much higher than the PTE + SMZ/GCE before elution. This is because the MIP film has more conductive sites and more [Fe(CN)_4_]^3−/4−^ permeation channels for electrochemical redox [35,36,37]. After rebinding of SMZ (Figure 3C, green line), the Δ*E*_p_ and *I*_p_ again increased and decreased compared with that of the MIP/GCE. In contrast, the CV curve of the NIP-SMZ/GCE (Figure 3D, green line) showed no difference from that of the NIP/GCE (Figure 3D, blue line). The above CV variation tendency is consistent with that of the EIS, and both demonstrate the successful preparation of the MIP film and its effective recognition toward the analyte SMZ.

### 3.4. Optimization of Sensor Performance

**Figure 4:**
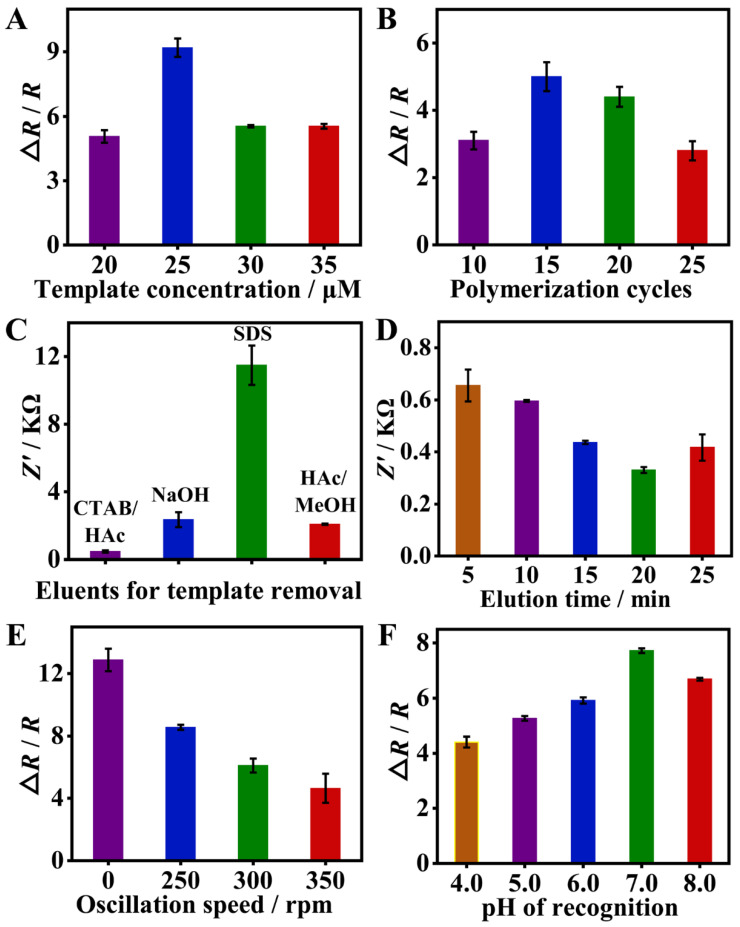
Optimization of experimental conditions, including template concentration (**A**), polymerization cycles (**B**), eluents and elution time for template removal (**C**,**D**), and oscillation speed and pH during recognition (**E**,**F**), to obtain a better sensing performance toward SMZ. Firstly, the eluent was optimized and determined by polymerization of 10 cycles at a concentration of 35 μM SMZ. Then, the concentration of SMZ was optimized in the case of polymerization of 10 cycles. Finally, the number of polymerization cycles was optimized at a template concentration of 25 μM. The rest are optimized in turn.

To enhance the sensor performance, various parameters affecting MIP fabrication and its recognition response were optimized. As shown in Figure 4A, with the increase in SMZ template concentration from 20 to 35 μM, the highest EIS response was achieved at 25 μM. This is because when the template concentration is too low, less SMZ is embedded in the polymer film, forming a small number of recognition sites and the EIS response is weak. On the contrary, when the concentration of SMZ is too high, complete removal of SMZ becomes more difficult and thus fewer recognition sites are created, which eventually leads to a weaker EIS response. Thus, 25 μM of the SMZ template was employed for the later study. The number of polymerization cycles controls the thickness of the imprinted film and can influence the removal and rebinding of the template [38]. As shown in Figure 4B, the EIS response of the sensor improved with the number of polymerization cycles from 10 to 15, but a further increase in the polymerization cycles to 20 and 25 resulted in a decrease in the EIS response to SMZ. Therefore, 15 cycles of polymerization were chosen as the optimal condition. For complete eluting of the SMZ template, several kinds of eluents were compared [39,40,41]. As shown in Figure 4C, CTAB/HAc is the most effective eluent with the lowest impedance value after elution. Furthermore, the elution time with CTAB/HAc was further optimized. As shown in Figure 4D, the impedance value of the MIP film decreased with the increase in elution time from 5 min to 20 min, indicating that the SMZ was eluted more cleanly. Yet, a further increase in elution time to 25 min resulted in a slight increase in impedance. Therefore, 20 min of elution with CTAB/HAc was eventually adopted.

Additionally, two factors that may affect SMZ recognition including oscillation speed and pH were optimized. As shown in Figure 4E, the faster the oscillation, the smaller the EIS response. Therefore, the MIP/GCE was finally incubated with SMZ under static conditions. The effect of pH is shown in Figure 4F, the EIS response increased with pH from 4.0 to 7.0, and then decreased from 7.0 to 8.0. Thus, pH 7.0 is chosen as the optimal condition for SMZ rebinding.

### 3.5. Quantitative Determination of SMZ

**Figure 5:**
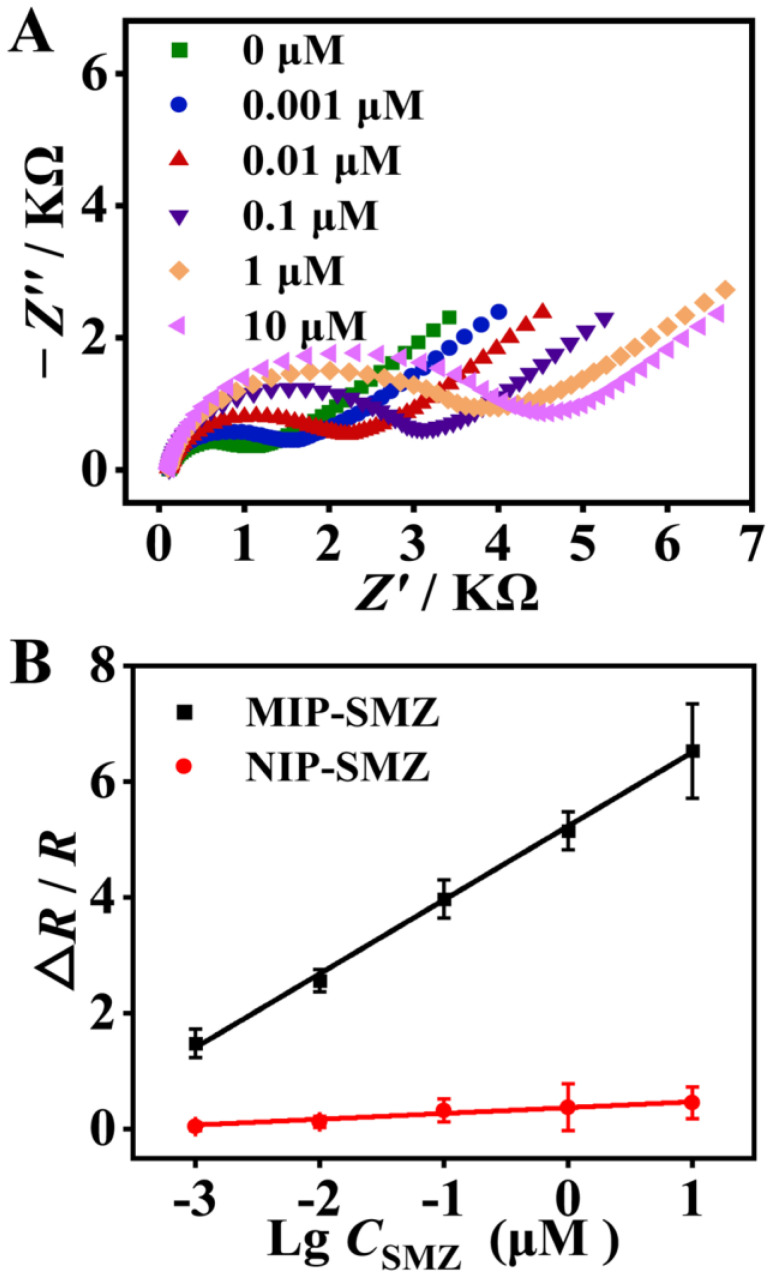
(**A**) Impedance spectra of the proposed sensor for detecting sulfamethizole (SMZ) from 0 to 10 μM by a 10-fold serial dilution. (**B**) Calibration curve of the EIS response versus logarithmic concentration of SMZ from 0.001 to 10 μM.

As shown in Figure 5A, with an increase in SMZ concentration from 0.001 to 10 μM, the impedance of the modified electrodes gradually increased. This is because the captured SMZ molecules partially blocked the electron transfer between the [Fe(CN)_6_]^3−/4−^ redox probe and the electrode surface, and increased the film impedance. Meanwhile, in a concentration ranging from 0.001 to 10 μM, the EIS response varied linearly with the logarithm concentration of SMZ (Figure 5B). The linear regression equation for this sensor was expressed as ∆*R*/*R* = 1.27lgC + 5.23 (*R*^2^ = 0.999). The LOD was calculated as 0.18 nM according to the 3σ/S rule (σ is the standard deviation of the blank sample (*n* = 10), and S is the slope of the calibration curve). In contrast, the EIS response of the NIP/GCE increased very slightly with the SMZ concentration. The results again confirmed the excellent recognition capability of the MIP toward SMZ. The LOD of the proposed sensor is lower or comparable to most previous MIP-based electrochemical sensors for detecting SAs antibiotics (Table S1) [42,43,44]. In contrast to the listed sensors that perform signal amplification through various nanomaterials with long-time and laborious synthesis processes, the fabrication of the MIP sensing interface in this study is much faster, and more eco-friendly, and can be completed within 30 min without use of any toxic organic solvents.

### 3.6. Performances of the Sensor

**Figure 6:**
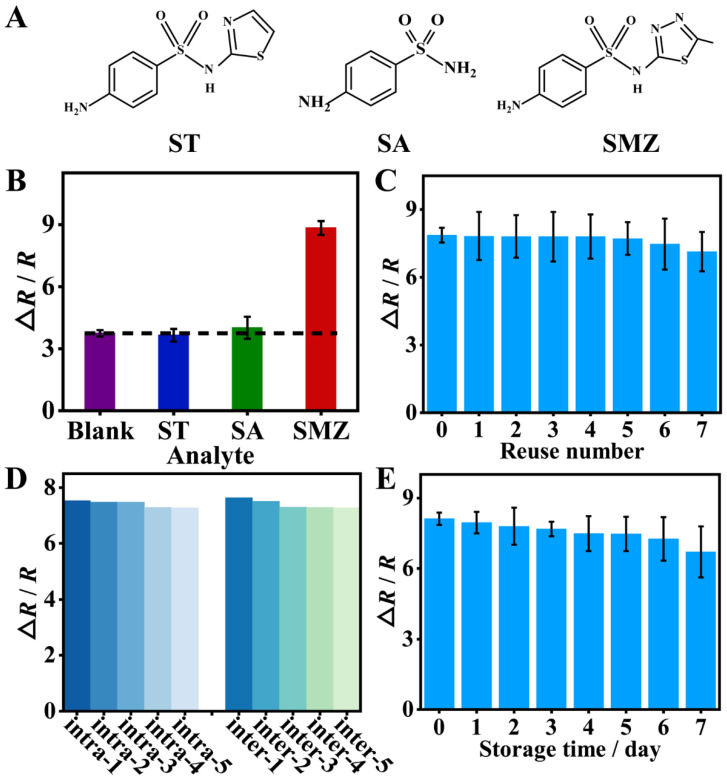
(**A**) Structural formula of sulfathiazole (ST), sulfanilamide (SA), and sulfamethizole (SMZ). (**B**) Selectivity: EIS response of the sensor to ST, SA, and SMZ at the same concentration. (**C**) Reusability: EIS response of the sensor to SMZ (0.1 μM) with different reuse numbers. (**D**) EIS response of the MIP sensor toward the intra-assay and inter-assay measurements of 0.1 μM SMZ. (**E**) The storage stability of the MIP/GCE.

The selectivity of the sensor was studied by selecting ST, and SA as interfering antibiotics based on their structure similarity (Figure 6A) to the target SMZ or the possibility of being in the same environment as SMZ. Both the target and interfering antibiotics were kept at the same concentration. As shown in Figure 6B, the EIS response for the interfering antibiotics, ST and SA, was almost the same as for the blank solution. However, for the target SMZ, the EIS response was approximately 2-fold greater than that for ST and SA. The results indicated that the sensor had good selectivity for SMZ due to the formation of specific recognition sites in the imprinted film.

The reusability was investigated by comparing the EIS response of the sensor after being reused at different times. By washing off the rebound SMZ with CTAB/HAc for 10 min, the MIP film could be effectively regenerated. As shown in Figure 6C, even after regeneration for 7 times, there were more than 90% of the initial determination signal, indicating the good reusability of the sensor.

The reproducibility of the sensor was assessed based on the intra-assay and inter-assay measurements of 0.1 μM SMZ (*n* = 5). The relative standard deviation was 1.47% for intra-assay and 2.01% for inter-assay (Figure 6D). Obviously, the sensor holds good reproducibility. In addition, the storage stability of MIP film-modified electrodes was evaluated based on the EIS detection of 0.1 μM SMZ. As can be seen from Figure 6E, the ∆*R*/*R* value decreased by 9.77% after 7 days of storage at 4 °C, indicating that the MIP film-modified electrode has good stability.

### 3.7. Real Samples Analysis

Water and milk samples were chosen to evaluate the feasibility of the MIP sensor for real samples analyses. The results are shown in Table 1. The average recoveries of SMZ ranged from 91.69% to 108.32%. For both samples, the minimum determinable concentration can be down to 1 nM which is much lower than the maximum residue limits (MRL) of sulfonamides in animal-source food set by the European Union (100 ppb, equivalent to approximately 0.37 μM for SMZ) [45]. The results suggested that the sensor had good practical applicability.

## 4. Conclusions

We demonstrated a simple and efficient sensing platform based on an electropolymerized MIP layer for direct detection of SMZ by the EIS. The results imply that a better recognition effect of the MIP can be achieved by the rational design of functional monomers. Compared to most previous molecularly imprinted electrochemical sensors for SAs (Table S1), a noteworthy advantage of the sensor is the rapid and green preparation of the MIP film within 30 min without use of any organic solvents. Moreover, the imprinted film can be regenerated for 7 times, which is beneficial to save assay cost and time. This sensor is expected to provide a simple, and facile method for the rapid, sensitive, and low-cost determination of other organic molecules. Yet, there are still difficulties in detecting analytes in complex matrices without complex sample pretreatments.

## Figures and Tables

**Table 1:** Determination of sulfamethizole (SMZ) in real samples (*n* = 3).

Sample	Added	Found	Recovery
	(nM)	(Mean ± SD/nM)	(%)
Water	0	0	/
	1	0.93 ± 0.21	92.97
	10	9.17 ± 0.18	91.69
	100	91.69 ± 0.09	98.75
Milk	0	0	/
	1	0.93 ± 0.21	92.97
	10	9.17 ± 0.38	91.69
	100	108.32 ± 0.22	108.32

## Data Availability

Date is contained within the article.

## Supplementary Materials

The following supporting information can be downloaded at: https://www.mdpi.com/article/10.3390/foods12081693/s1, Figure S1: The optimized structure for several monomers: (A) 3-thiopheneacetic acid (TAA), (B) 3-thiopheneethanol (TE), (C) 3-thiophenemethylamine (TM), (D) 3-thiopheneboronic acid (TBA),and (E) sulfamethizole (SMZ) molecule; Figure S2: Optimized geometric structures of several binding modes of the complexes between SMZ and TAA (A–C) or TE (D–F); Figure S3: Optimized geometric structures of several binding modes of the complexes between SMZ and TM (A–C) or TBA (D–F); Figure S4: The equivalent circuit model, where Rs, Cdl, and Zw represent solution resistance, double-layer capacitance, and Warburg impedance, respectively; Figure S5: (A–D) Electrochemical impedance spectroscopy (EIS) for each electropolymerized 3-substituted thiophene in the presence and absence of SMZ template, and the corresponding MIP film after template removal. (E–H) EIS response of each MIP and NIP towards target SMZ; Table S1: Comparison of the proposed sensor with other reported molecularly imprinted electrochemical sensors for detecting sulfonamide antibiotics [27,28,29,42,43,44,46,47,48,49,50,51].
