# Supplementary material for: Rapid and Sensitive Detection of Sulfamethizole Using a Reusable Molecularly Imprinted Electrochemical Sensor
Source: Foods. 2023 Apr 19;12(8):1693. doi: 10.3390/foods12081693 (PMC10137692; doi:10.3390/foods12081693)
Supplement: Supplementary file 1 [file foods-12-01693-s001.zip › foods-2337703-supplementary.pdf]

Rapid and sensitive detection of sulfamethizole using a reusable molecularly imprinted electrochemical sensor

Jie Kong [1], Xiaoli Xu [1], Yixin Ma [1], Junjian Miao [1,*] and Xiaojun Bian [1,2,3,*]

1 College of Food Science and Technology, Shanghai Ocean University, Shanghai 201306, China; m200300916@st.shou.edu.cn (J.K.); xlxu981023@163.com (X.X.)

2 Laboratory of Quality and Safety Risk Assessment for Aquatic Product on Storage and Preservation

(Shanghai), Ministry of Agriculture, Shanghai 201306, China

3 Shanghai Engineering Research Center of Aquatic-Product Processing & Preservation, Shanghai 201306, China

* Correspondence: jjmiao@shou.edu.cn (J.M.); xjbian@shou.edu.cn (X.B.)

Quantum chemical calculations

As the template molecule has multiple functional groups and has a certain flexibility, there will be multiple binding modes when they are combined. In order to obtain the low-lying binding mode of the system, we used the following scheme for conformational search. For the heterodimers formed by each monomer and template molecule, we created five different initial structures according to the possible binding sites and then used molecular dynamics for conformational sampling. The specific conditions of molecular dynamics (MD) are temperature=400 K, simulation time=100 ps, step size=2.0 fs, dumptime=50 fs, and other default values. In order to enhance the sampling effect, the temperature was set slightly higher than the normal temperature. The software used for MD calculations is xtb (version 6.3.2) [1]. For the ~10000 structures obtained, we use Crest to optimize them, and the level is gfn1 [2, 3]. For the optimized structure, deduplication is carried out according to the difference in energy and structure, and the program used is isostat [4]. The deduplication threshold for energy is 0.25 kcal/mol, and the structure deduplication threshold is 0.1Å. In order to obtain more accurate structures, the obtained structures were optimized using the B97-3c method, and was deduplicated further using isostat. For these final structures, in order to obtain more accurate electronic energies, the single-point energies were calculated using PWPB95(D3-BJ)/def2-QZVPP. The calculation programs used in the last two steps are orca [5] and molclus [4]. For monomers and template molecules, the search is performed in the same way.

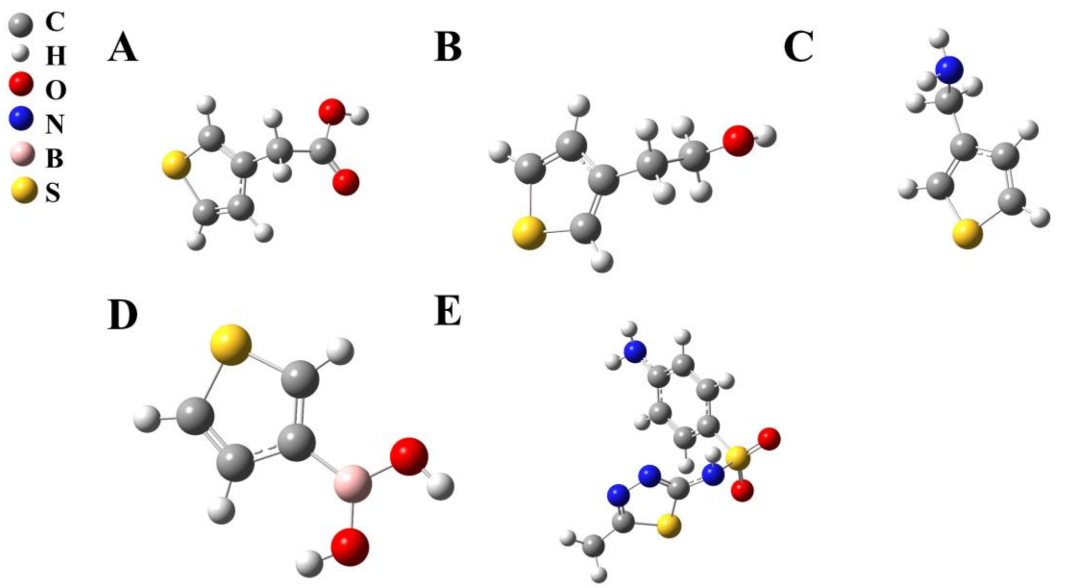

Figure. S1. The optimized structure for several monomers: (A) 3-thiopheneacetic acid (TAA), (B) 3-thiopheneethanol (TE), (C) 3-thiophenemethylamine (TM), (D) 3-thiopheneboronic acid (TBA), and (E) sulfamethizole (SMZ) molecule.

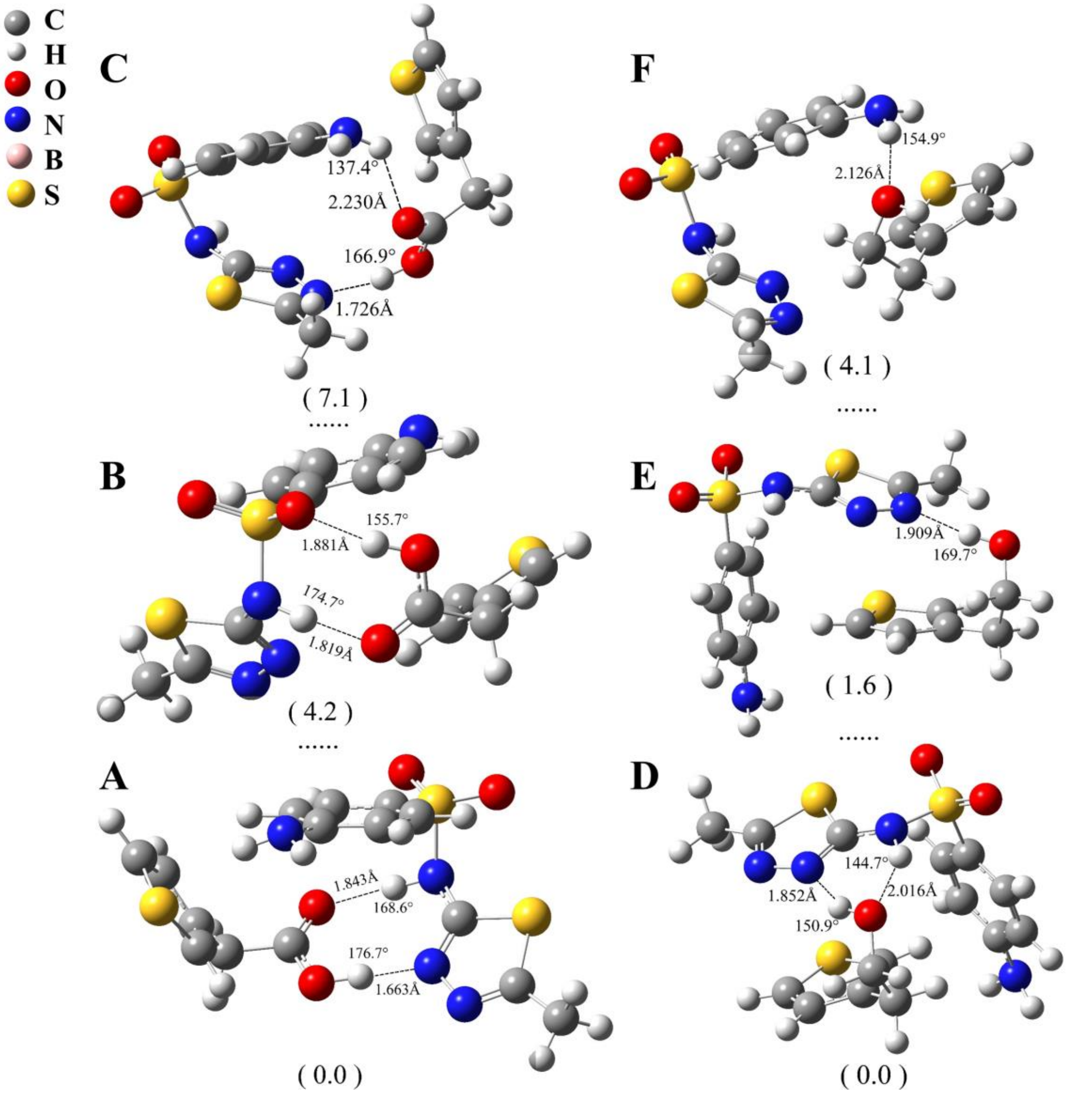

Figure. S2. Optimized geometric structures of several binding modes of the complexes between SMZ and TAA (A-C) or TE (D-F). Fig. S2 A, D is the lowest energy structures as shown in Fig. 1A and 1B. The presence of hydrogen bonds is indicated by the light black dashed lines. The values near the hydrogen bonds are the bond lengths and bond angles, and values in brackets are energies (unit: kcal/mol) relative to that of lowest energy structure. The ellipses indicate that there are many similar structures (the system below them), and their differences in energy and structure are very small, so only one representative structure was shown. Level of theory: PWPB95(D3-BJ)/def2-QZVPP//B97-3c.

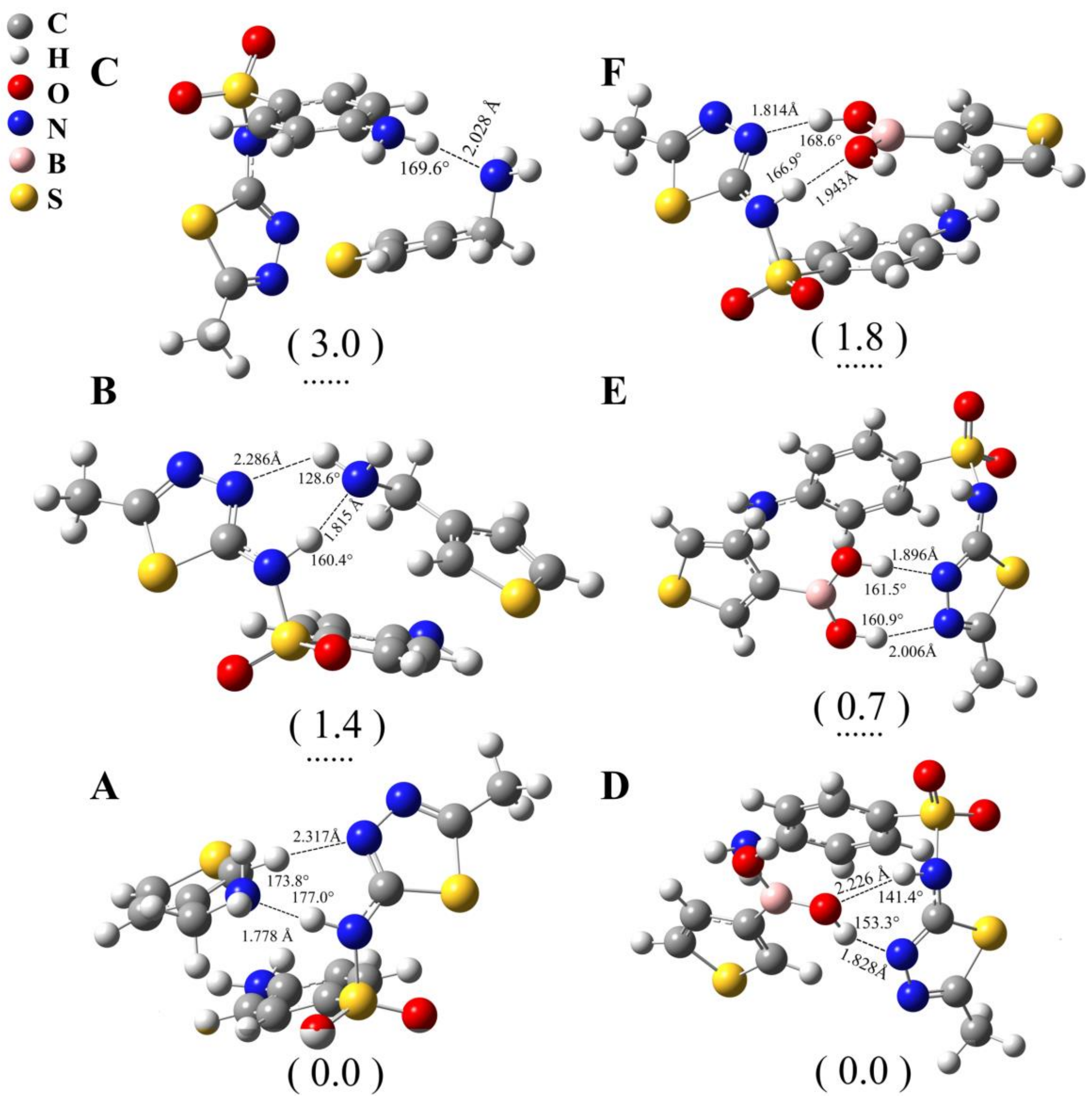

Figure. S3. Optimized geometric structures of several binding modes of the complexes between SMZ and TM (A-C) or TBA (D-F). Fig. S3 A, D is the lowest energy structures as shown in Fig. 1C and 1D. The presence of hydrogen bonds is indicated by the light black dashed lines. The values near the hydrogen bonds are the bond lengths and bond angles, and values in brackets are energies (unit: kcal/mol) relative to that of lowest energy structure. The ellipses indicate that there are many similar structures (the system below them), and their differences in energy and structure are very small, so only one representative structure was shown. Level of theory: PWPB95(D3-BJ)/def2-QZVPP//B97-3

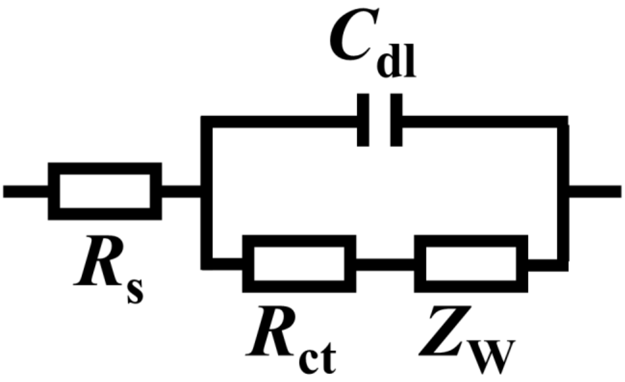

Figure. S4. The equivalent circuit model, where R_s, C_{dl}, and Z_w represent solution resistance, double-layer capacitance, and Warburg impedance, respectively.

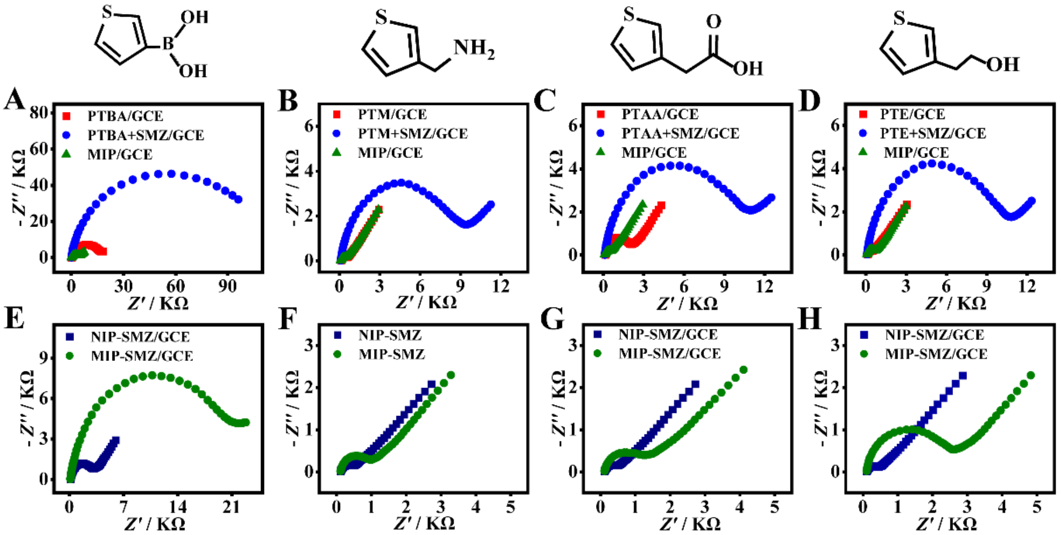

Figure. S5. (A-D) Electrochemical impedance spectroscopy (EIS) for each electropolymerized 3-substituted thiophene in the presence and absence of SMZ template, and the corresponding MIP film after template removal. (E-H) EIS response of each MIP and NIP towards target SMZ.

Table S1. Comparison of the proposed sensor with other reported molecularly imprinted electrochemical sensors for detecting sulfonamide antibiotics.

Sensor	Target	linear range (μM)	LOD (nM)	Sensor fabrication	Ref.
PDA-MIP/GE	sulfamethoxazole	8.0×10^{-1}-1.7×10^{2}	8×10^{2}	＞2.5 h	[6]
MIP/Ni(OH)$_2$ /NF	sulfapyridine	5.9×10^{-1}-1.34×10^{3}	3.57×10^{2}	＞11 h	[7]
MIP/MoS$_2$ /NH$_2$-MWCNT@COF/GCE	sulfamerazine	3.0×10^{-1}-2.0×10^{2}	1.1×10^{2}	＞29 h	[8]
MIP/SPE	sulfamethizole	12.8×10^{-2}-1.6	9×10^{-1}	＞16 h	[9]
MIP/GE	sulphanilamide	5.8×10^{-3}-7.18×10^{-1}	5.8×10^{-1}	about 30 min	[10]
MIP/CuS/Au@COF/GCE	sulfathiazole	$1.0\text{x}10^{-5}$-1×10^{2}	4.3×10^{-3}	＞30 h	[11]
MIPs/UnZ-CCTF	sulfaquinoxaline	1.0×10^{-7}-2×10^{-6}	7.6×10^{-4}	＞27h	[12]
MIP/GCE	sulfamethizole	1.0×10^{-3}-1.0×10^{1}	1.8×10^{-1}	about 35min	This work

MIP: molecularly imprinted polymer; **GE**: Gold electrode; **PDA**: polydopamine; **Ni(OH)$_2$/NF**: Ni(OH)$_2$nanoarrays in situ grown on nickel foam; **GCE**: glassy carbon electrode; **CuS**: copper sulfide; **MoS$_2$**: disulfide; **NH$_2$-MWCNT**: amino-functionalized carbon nanotubes; **COF**: covalent organic frameworks; **H-GNs**: hybrid nanosheets; **SPE**: Screen-Printed Electrode; **UnZ-CCTF**: unzipped covalent triazine framework; **3D**: Three-dimensional.

References

[1] S. Spicher, S. Grimme, Robust Atomistic Modeling of Materials, Organometallic, and Biochemical Systems, Angew Chem Int Edit 59(36) (2020) 15665-15673.

[2] P. Pracht, F. Bohle, S. Grimme, Automated exploration of the low-energy chemical space with fast quantum chemical methods, Phys Chem Chem Phys 22(14) (2020) 7169-7192.

[3] S. Grimme, Exploration of Chemical Compound, Conformer, and Reaction Space with Meta-Dynamics Simulations Based on Tight-Binding Quantum Chemical Calculations, J Chem Theory Comput 15(5) (2019) 2847-2862.

[4] T. Lu, Molclus program, Version 1.9, http://www.keinsci.com/research/molclus.html (accessed 12 18, 2022).

[5] F. Neese, Software update: the ORCA program system, version 4.0, WIREs Computational Molecular Science 8(1) (2017).

[6] A. Turco, S. Corvaglia, E. Mazzotta, P.P. Pomba, C. Malitesta, Preparation and characterization of molecularly imprinted mussel inspired film as antifouling and selective layer for electrochemical detection of sulfamethoxazole, Sensor Actuat B-Chem 255 (2018) 3374-3383.

[7] Z.Y. Liu, Y. Zhang, J.H. Feng, Q.Z. Han, Q. Wei, Ni(OH)2 nanoarrays based molecularly imprinted polymer electrochemical sensor for sensitive detection of sulfapyridine, Sensor Actuat B-Chem 287 (2019) 551-556.

[8] Y.F. Sun, L.H. Xu, G.I.N. Waterhouse, M.L. Wang, X.G. Qiao, Z.X. Xu, Novel three-dimensional electrochemical sensor with dual signal amplification based on MoS2 nanosheets and high-conductive NH2-MWCNT@COF for sulfamerazine determination, Sensor Actuat B-Chem 281 (2019) 107-114.

[9] A.G. Ayankojo, J. Reut, A. Opik, V. Syritski, Sulfamethizole-imprinted polymer on screen-printed electrodes: Towards the design of a portable environmental sensor, Sensor Actuat B-Chem 320 (2020) 128600-128609.

[10] A.K. Prusty, S. Bhand, Molecularly Imprinted Polyresorcinol Based Capacitive Sensor for Sulphanilamide Detection, Electroanal 31(9) (2019) 1797-1808.

[11] Y.F. Sun, H.J. Gao, L.H. Xu, G.I.N. Waterhouse, H.Y. Zhang, X.G. Qiao, Z.X. Xu, Ultrasensitive determination of sulfathiazole using a molecularly imprinted electrochemical sensor with CuS microflowers as an electron transfer probe and Au@COF for signal amplification, Food Chemistry 332 (2020) 127376.

[12] X.H. Ma, S.H. Li, C.H. Pang, Y.H. Xiong, J.P. Li, A Cu(II)-anchored unzipped covalent triazine framework with peroxidase-mimicking properties for molecular imprinting-based electrochemiluminescent detection of sulfaquinoxaline, Microchim Acta 185(12) (2018) 546.
